# Physiological responses and transcriptome analysis of soybean under gradual water deficit

**DOI:** 10.3389/fpls.2023.1269884

**Published:** 2023-10-26

**Authors:** Yuwen Xu, Di Song, Xingliang Qi, Muhammad Asad, Sui Wang, Xiaohong Tong, Yan Jiang, Shaodong Wang

**Affiliations:** ^1^ Northeast Agricultural University, Agricultural College, Harbin, China; ^2^ Heilongjiang Academy of Green Food Science/National Soybean Engineering Technology Research Center, Harbin, China

**Keywords:** drought stress, soybean, photosynthesis, stomata, water use efficiency, transcriptome

## Abstract

Soybean is an important food and oil crop widely cultivated globally. However, water deficit can seriously affect the yield and quality of soybeans. In order to ensure the stability and increase of soybean yield and improve agricultural water use efficiency (WUE), research on improving drought tolerance and the efficiency of water utilization of soybeans under drought stress has become particularly important. This study utilized the drought-tolerant variety Heinong 44 (HN44) and the drought-sensitive variety Suinong 14 (SN14) to analyze physiological responses and transcriptome changes during the gradual water deficit at the early seed-filling stage. The results indicated that under drought conditions, HN44 had smaller stomata, higher stomatal density, and lower stomatal conductance (Gs) and transpiration rate as compared to SN14. Additionally, HN44 had a higher abscisic acid (ABA) content and faster changes in stomatal morphology and Gs to maintain a dynamic balance between net photosynthetic rate (Pn) and Gs. Additionally, drought-tolerant variety HN44 had high instantaneous WUE under water deficit. Further, HN44 retained a high level of superoxide dismutase (SOD) activity and proline content, mitigating malondialdehyde (MDA) accumulation and drought-induced damage. Comprehensive analysis of transcriptome data revealed that HN44 had fewer differentially expressed genes (DEGs) under light drought stress, reacting insensitivity to water deficit. At the initial stage of drought stress, both varieties had a large number of upregulated DEGs to cope with the drought stress. Under severe drought stress, HN44 had fewer downregulated genes enriched in the photosynthesis pathway than SN14, while it had more upregulated genes enriched in the ABA-mediated signaling and glutathione metabolism pathways than SN14. During gradual water deficit, HN44 demonstrated better drought-tolerant physiological characteristics and water use efficiency than SN14 through key DEGs such as *GmbZIP4*, *LOC100810474*, and *LOC100819313* in the major pathways. Key transcription factors were screened and identified, providing further clarity on the molecular regulatory pathways responsible for the physiological differences in drought tolerance among these varieties. This study deepened the understanding of the drought resistance mechanisms in soybeans, providing valuable references for drought-resistant soybean breeding.

## 1 Introduction

Soybean (*Glycine max* L.) is a globally cultivated oilseed crop and serves as a significant source of protein and fat (Liu et al., 2020). As an important source of protein and edible oil, soybean consumption demand has skyrocketed, and the supply and demand gap is widening. Therefore, improving soybean yield and increasing benefits are still important directions for soybean research. However, due to global climate change and increasing water scarcity, drought has emerged as one of the primary abiotic stress factors, which inhibits the potential for a high yield of soybeans (Lian et al., 2021). Thus, it is urgent to study the stress tolerance and resistance of soybeans to enhance their ability to cope with drought.

Drought has a range of impacts on plants, encompassing alterations in the internal structure of leaves and photosynthesis, variation in osmotic regulation systems, disruption of hormone homeostasis, production of reactive oxygen species (ROS), and damage to cell membranes (Xu et al., 2010). Drought stress inhibits leaf photosynthesis by affecting stomatal movement, resulting in reduced photosynthetic rate, stomatal conductance, and intercellular CO_2_ concentration. Therefore, plants have evolved complex physiological and molecular mechanisms to adapt to drought stress (Chimungu et al., 2014). Phytohormones, particularly abscisic acid (ABA), play a pivotal role in defending against abiotic stresses (Vishwakarma et al., 2017). ABA levels significantly escalate under water-deficient conditions, and ABA receptors PYR1/PYL/RCAR bind to ABA and target protein phosphatase type 2C (PP2C). This release activates the kinase activity of Snf1-related kinase 2 (SnRK2), which then phosphorylates downstream transcription factors and transporters to activate ABA reactions and improve plant stress resistance (Ohashi et al., 2006; Wang et al., 2021). Plants integrate multiple environmental signals to optimize stomatal aperture and density in different environments to maintain high water use efficiency (WUE) (Bertolino et al., 2019). Research has demonstrated that targeted changes in stomatal development can improve the WUE and drought resistance of crop varieties (Yin et al., 2017). In response to drought stress, plants also activate antioxidant and osmoregulation systems, mitigating drought-induced damage by increasing antioxidant enzyme activity and osmoregulation protectants such as proline (Dien et al., 2019; Wang et al., 2022a). Additionally, the increase in superoxide dismutase (SOD) activity and peroxidase (POD) activity enhances the metabolism of glutathione, which can eliminate excessive ROS in the plant, inhibit the accumulation of malondialdehyde, and reduce the damage caused by drought (Zhang et al., 2020). These physiological changes are coordinated by drought-related functional and regulatory genes. Functional genes, such as those associated with photosynthesis and antioxidant enzymes, are direct response genes that respond to perceived micro-environmental changes under stress. Conversely, regulatory genes are indirect response genes that facilitate stress adaptation through signaling pathways. These genes include transcription factors and protein kinases (Shinozaki and Yamaguchi-Shinozaki, 2006). Some genes and metabolites have been demonstrated to play roles in drought regulation. For example, under drought stress, soybeans enhanced their prevention of reactive oxygen damage and tolerance to abiotic stress by upregulating or downregulating the expression of ascorbic acid and glutathione-related proteins at the protein level (Zhou et al., 2008). Additionally, some transcription factors have been confirmed to be the main players in water stress signals, including MYB, bHLH, AP2/EREBP, bZip, and NAC. These transcription factors respond to drought through various pathways, including signaling cell motility pathways and metabolic synthesis (Lata and Prasad, 2011; Baldoni et al., 2015; Joshi et al., 2016; Ariyarathne and Wone, 2022).

Various omics have shed light on the responses of plants to abiotic stresses (Basim et al., 2021). For instance, RNA sequencing (RNA-seq) is a high-throughput sequencing technology with high accuracy and low cost and has been widely used in gene expression analysis in plants for evaluating the interactions between plants and abiotic stresses (Zhang and Song, 2017). Yang et al (2023) conducted a transcriptome analysis of leaf tissues under drought stress in the drought resistance soybean SS-2 and the drought-sensitive soybean Taekwang, and they discovered a significant increase in the expression of phosphatidylinositol 4-phosphate 5-kinase (PIP5K), which is involved in lipid signaling pathways, in SS2-2 (Yang et al., 2023). Additionally, Wang et al (2022b) carried out transcriptome and metabolome analyses on the drought-resistance variety Heinong 44 (HN44) and the drought-sensitive variety Heinong 65 (HN65), and they discovered that in the soybean drought resistance pathway, the tricarboxylic acid (TCA) cycle was key in mediating processes such as hormone transport and amino acid synthesis (Wang et al., 2022b). However, the complex network regulation of soybeans requires further exploration to discover more genes for drought tolerance and water use efficiency.

Previously, much research has been conducted on the transcriptomics of soybeans under drought stress. However, most of these studies have been limited to individual varieties, with less emphasis on the differences in drought tolerance among different varieties. A study on HN44 and Suinong 14 (SN14) soybean varieties selected by Jiang et al. (2022) was conducted to understand their response mechanisms under gradual water deficit during the early seed-filling stage (Jiang et al., 2022). Thus, building on the aforementioned research background and research foundation of our research group, this study analyzed the physiology characteristics and transcriptome responses of different soybean varieties under water deficit conditions. Specifically, we aimed to identify key differentially expressed genes involved in regulatory pathways related to drought tolerance and explore potential transcription factors that could improve drought resistance and water use efficiency in soybeans. This study will improve our understanding of the physiological responses and molecular mechanisms of different soybean varieties under water deficit conditions.

## 2 Materials and methods

### 2.1 Plant materials and stress treatments

**Figure 1:**
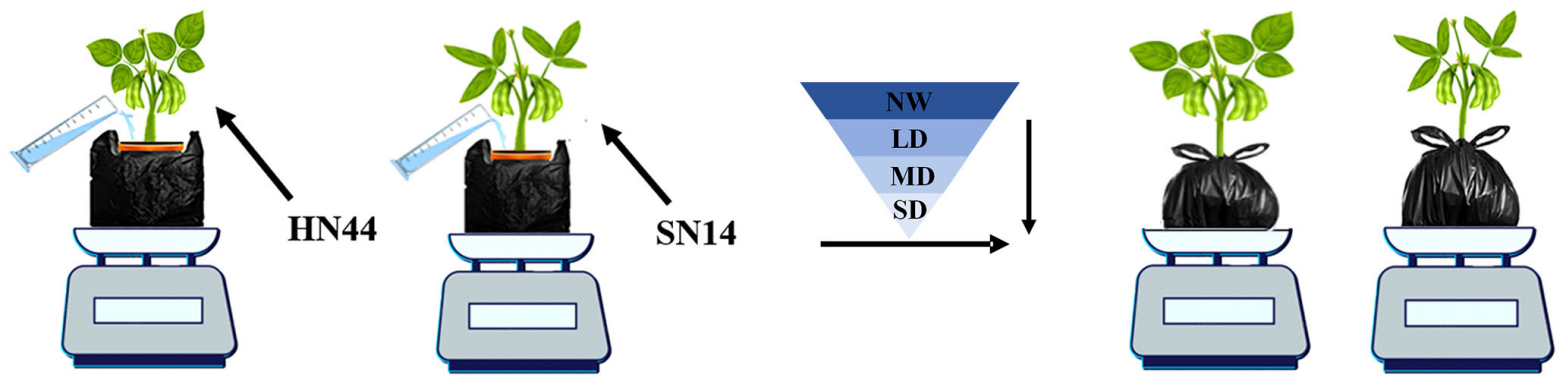
Gradual water deficit treatments. HN44, drought-tolerant variety Heinong 44; SN14, drought- sensitive Suinong 14; NW, normal water, 70%PSWC; LD, light drought, 60%PSWC; MD, moderate drought, 55%PSWC; SD, severe drought, 50%PSWC. The same as below.

In this study, the drought-tolerant variety HN44 and the drought-sensitive SN14 were tested in 2021 at the glass canopy of Northeast Agricultural University. Soybean seeds with full grains and uniform size were selected for sowing in 1.5-gallon pots with a dimension of 17.5 cm top diameter × 14 cm bottom diameter × 16 cm height with three biological replicates for each treatment. Before sowing, the weight of each empty pot was recorded. Subsequently, the soil in each pot was saturated with deionized water and allowed to drain overnight. The weight of the pot soil water capacity (PSWC) was the total weight of the soil mixture and water and was recorded and considered as 100% of the PSWC (100%PSWC) at that time (Bramley et al., 2022). Two seeds were sown in each pot, and one seedling was retained in each pot after germination. The pots were bundled with black plastic bags to prevent surface moisture evaporation and loss. From sowing to the early seed-filling stage, the pots’ weight was measured daily and watered to 70%PSWC to maintain normal plant moisture (Jiang et al., 2020). At the early stage of seed-filling (R5), a completely randomized experimental design consisted of drought treatment for each variety, and the PSWC of each pot was monitored and adjusted daily by weighing. Drought treatments included normal water (NW; 70%PSWC), light drought (LD; 60%PSWC), moderate drought (MD; 55%PSWC), and severe drought (SD; 50%PSWC), forming a gradual water deficit. Each water deficit treatment lasted for 1 day (**Figure 1**).

### 2.2 Sampling and measurement

#### 2.2.1 Photosynthetic characteristics

After treatment, the third fully expanded trifoliate leaves at the tip of the main stem were measured. These measurements were conducted between 8:00 AM and 10:00 AM on a sunny day using a Cl-340 photosynthesizer (CID Bio-Science, Inc., Camas, WA, USA). The photosynthetic characteristics assessed included net photosynthetic rate (Pn), stomatal conductance (Gs), transpiration rate (Tr), and intercellular carbon dioxide concentration (Ci), with five replicates for each treatment. The instantaneous water use efficiency (WUE_i_) was calculated as the ratio of Pn to Tr.


$$\text{WUEi}=\frac{\text{Pn}}{\text{Tr}}$$

#### 2.2.2 Stomatal parameters

Blotting and microscopy techniques were employed to visualize stomatal characteristics. On the abaxial surface of the fourth fully expanded trifoliate leaves at the tip of the main stem, five 0.5-cm^2^ areas were coated with transparent nail polish. After the nail polish dried, the stomatal characteristics of the leaves in different treatments were observed with a fluorescence microscope (Olympus BX53, Tokyo, Japan); the stomatal length (SL), stomatal width (SW), area of the field of view (S), and number of stomata in each field of view (Q) (0.5 mm × 0.5 mm) were measured; stomatal density (SD_L_) was calculated based on the above indexes. Ten replicates were performed for each treatment..


$$\text{SDL}=\frac{\;}{\text{QS}}$$

#### 2.2.3 Abscisic acid content

A frozen sample with a mass of 0.1 g (the second fully expanded trifoliate leaves at the tip of the main stem) was taken and added to 0.9 ml of phosphate-buffered saline (PBS) solution with a concentration of 0.01 mol/L for ice bath grinding. The supernatant was collected after centrifugation at 4,000 rpm for 15 min at 4°C. Then, using the Plant Hormone Abscisic Acid ELISA kit (Shanghai Enzyme Link Biotechnology, Shanghai, China), ABA concentration was measured.

#### 2.2.4 Leaf relative water content

After treatment, the third fully expanded trifoliate leaves at the tip of the main stem were harvested, and their fresh weight was recorded. The leaves were immersed in water and placed in a dark room overnight. Afterward, the excess surface moisture on the leaves was removed using filter paper, the saturated weight was measured, and the leaves were then carefully placed in an 80°C oven and dried until a constant weight was achieved, at which point the dry weight was recorded.


$$\text{RLWC}=\frac{\left(\text{FW}-\text{DW}\right)}{\left(\text{SQ}-\text{DW}\right)}$$

#### 2.2.5 Determination of superoxide dismutase activity, malondialdehyde content, and proline content

The SOD activity, malondialdehyde (MDA) content, and proline content were measured using the SOD Activity Assay Kit (SOD-1-W, Comin, Hangzhou, China), MDA Quantitative Assay Kit (MDA-1-Y, Comin), and Proline Quantitative Assay Kit (PRO-1-Y, Comin), respectively. All testing and analyses were conducted according to the manufacturer’s instructions.

#### 2.2.6 RNA extraction, library construction, and sequencing

Based on the physiological analysis results under different treatments, transcriptome sequencing was performed on the leaves subjected to LD, SD, and NW conditions. After 1 day of stress treatment, the first fully expanded trifoliate leaves at the tip of the main stem were taken as samples for total plant RNA extraction. The plant total RNA was extracted using the RNAprep Pure Plant Kit (Tiangen, Beijing, China) according to the instructions provided by the manufacturer. RNA quality identification, library construction, and sequencing were performed by Frasergen (Wuhan, China).

#### 2.2.7 RNA-seq analysis and gene expression analysis

Clean reads were compared to the Wm82.a2.v1 reference genome using Hisat2 (v2.2.1) software, and the number of reads compared to each gene was counted, using transcripts per thousand bases per million mapped reads transcripts (TPM) values per thousand bases to determine gene expression levels. The transcriptome raw sequencing data from this study were submitted to the National Center for Biotechnology Information (NCBI) (http://www.ncbi.nlm.nih.gov/) database as individual BioProjects: PRJNA1011827. Differentially expressed genes (DEGs) were screened using DESeq2 software, and Padj< 0.01 and |log_2_FoldChange ≥ 2| were used as the differential gene screening conditions. Differentially communicated qualities were explained to the Gene Ontology (GO) and Kyoto Encyclopedia of Genes and Genomes (KEGG) pathway datasets to acquire comments on the data.

#### 2.2.8 Quantitative real-time PCR analysis

Total RNA extracted from the two leaves of soybean plants 1 day after the seed-filling period (R5) was isolated with total RNA using the total RNA rapid extraction kit (BioTeke Corporation, Beijing, China) and reverse transcribed using PrimeScript™ RT reagent Kit (TaKaRa, Mountain View, CA, USA). The qRT-PCR was performed on an AriaMx quantitative PCR instrument using SYBR Premix Ex Taq™ (TaKaRa), and each reaction was performed in triplicate. The actin gene *Glyma.02G091900* was used as the internal reference (Liu et al., 2016). Gene relative expression levels were calculated according to the 2^−ΔΔCT^ method. The primer sequences are shown in **Supplementary Table 1.**

#### 2.3 Statistical analysis

All data were recorded in Excel 2016 (Microsoft Corp., Redmond, WA, USA). Analysis of variance (ANOVA) for relative leaf water content (RLWC), ABA content, photosynthetic parameters, and stomatal parameters was performed using SPSS 26.0 (IBM Corp., Armonk, NY, USA). Significance among gradual water deficit treatments was determined by the least significant difference (LSD) test, *p*< 0.05. GO enrichment analysis with KEGG enrichment analysis of transcriptome data was performed with R software (4.2.2). Figures were plotted using Origin 2021pro (OriginLab Co., Northampton, MA, USA).

## 3 Results

### 3.1 Effects of gradual water deficit on physiological traits

#### 3.1.1 Effect of gradual water deficit on photosynthetic indexes

**Figure 2:**
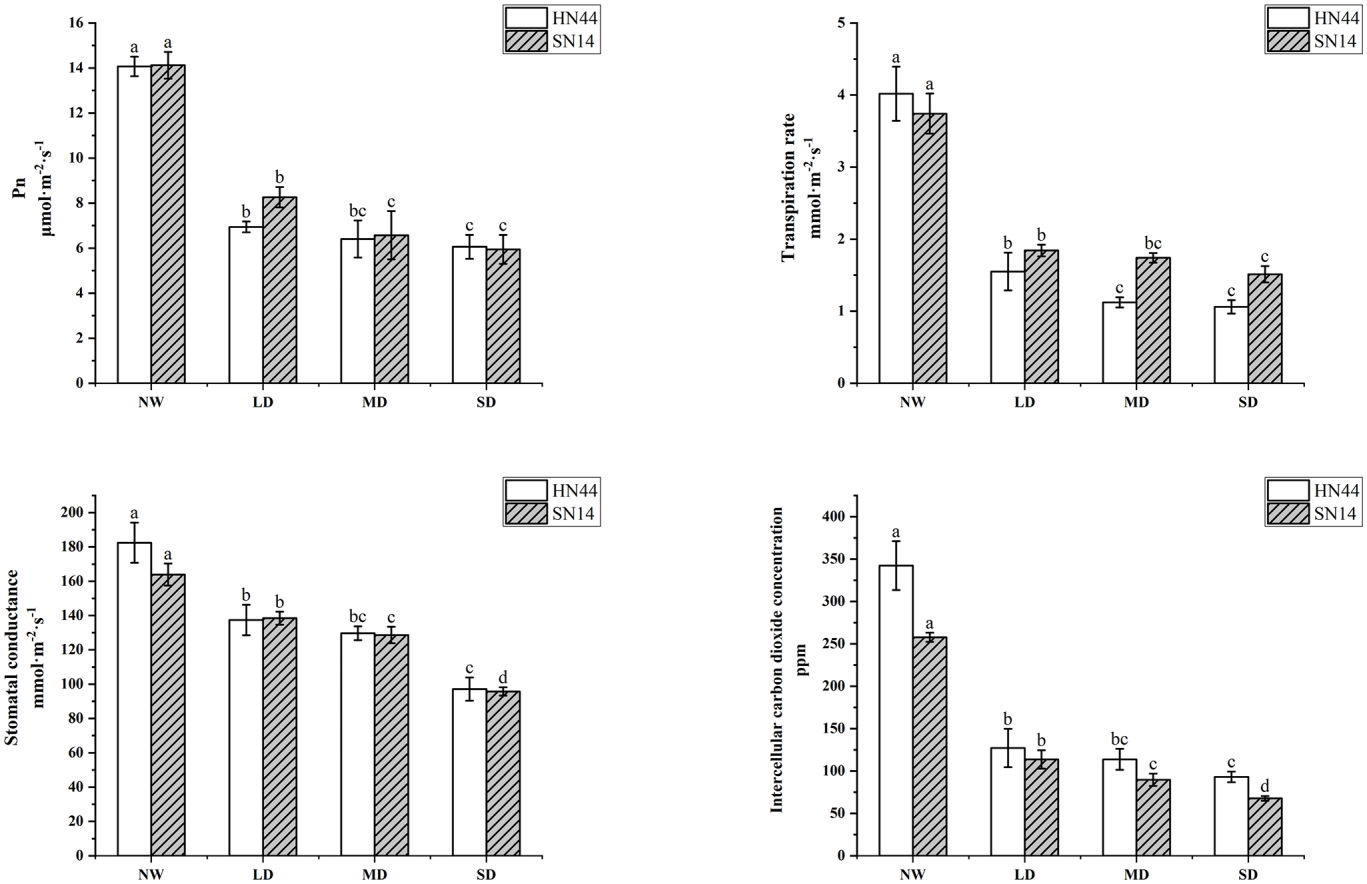
Effect of gradual water deficit on photosynthetic parameters. Data indicate means ± standard deviation with five replications. Different letters for the same variety indicate significant differences between treatments as tested by LSD at *p*< 0.05. LSD, least significant difference.

The Pn, Tr, Gs, and Ci of HN44 and SN14 all decreased with water stress increasing (**Figure 2**). Each treatment was significantly different from the control (*p*< 0.05). Compared to NW, Pn respectively decreased by 50.67%, 54.51%, and 56.92% for HN44 and by 41.50%, 53.47%, and 57.93% for SN14 under LD, MD, and SD conditions; Tr respectively decreased by 61.44%, 72.14%, and 73.63% for HN44 and by 50.8%, 53.48%, and 59.62% for SN14 under LD, MD, and SD conditions. The greater decrease in Tr for HN44 under gradual water stress reduced water loss and improved drought resistance. Similarly, Gs respectively decreased by 24.71%, 28.91%, and 46.74% for HN44 and by 15.52%, 21.51%, and 41.58% for SN14 under LD, MD, and SD conditions; Ci respectively decreased by 60.98%, 65.29%, and 66.24% for HN44 and by 55.91%, 65.23%, and 73.75% for SN14 under LD, MD, and SD conditions. The larger decrease in Gs for HN44 reduced water transpiration by rapidly reducing Gs. The reduction in Gs caused a decrease of Ci, which conversely inhibited the assimilation of CO_2_ and reduced its photosynthetic capacity. As the stress severity increased, the photosynthetic parameters of both varieties were suppressed to a certain extent, affecting the photosynthetic system of soybean plants. Pn, Gs, and Tr of HN44 decreased greater than those of SN14 under different water stress treatments; HN44 rapidly adjusted its photosynthetic gas channels, changed gas parameters, reduced water transpiration, and maintained its water status.

#### 3.1.2 Effect of gradual water deficit on stomatal characteristics

**Figure 3:**
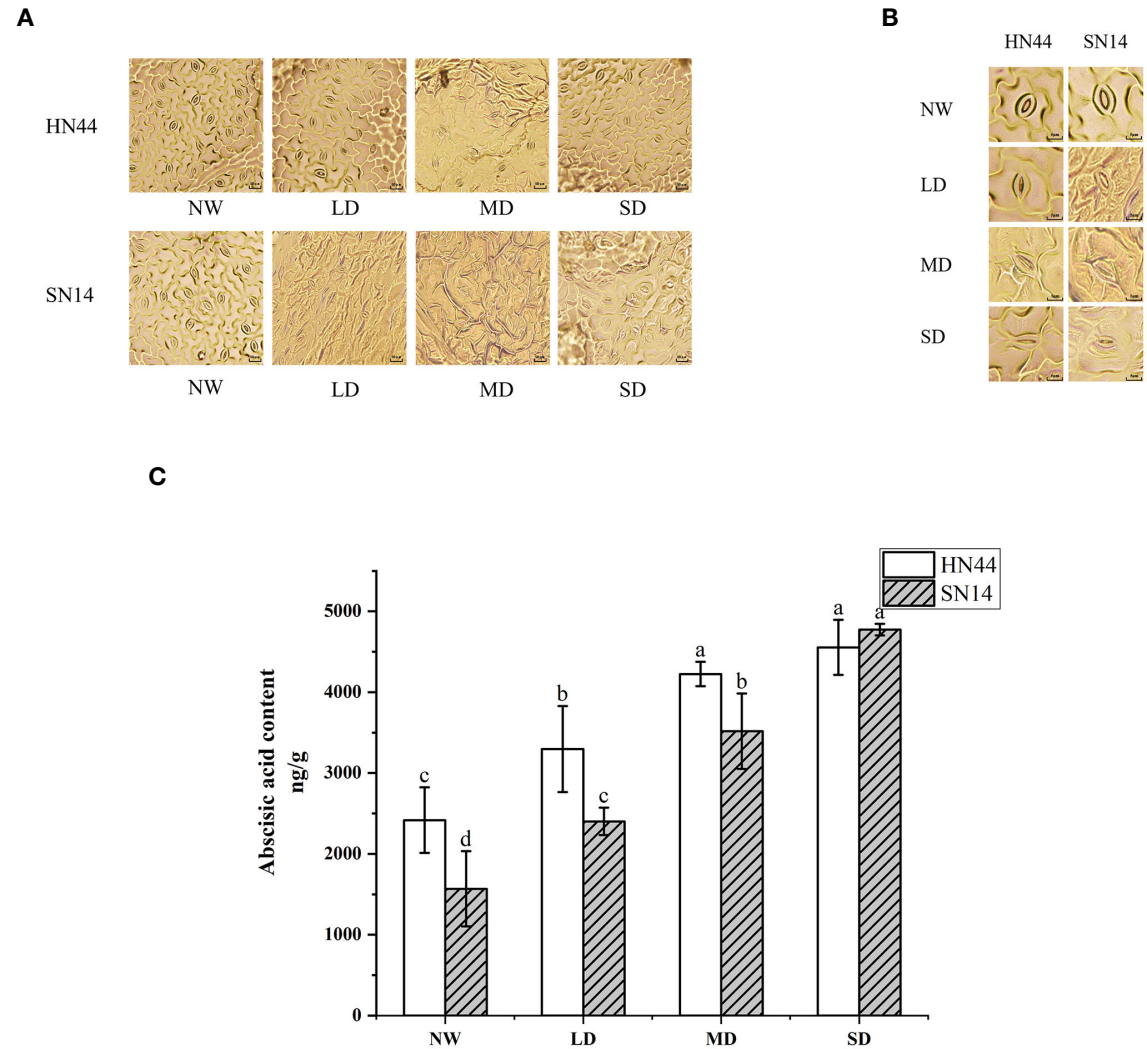
Effect of gradual water deficit on stomatal characteristics: **(A)** standard scale was 50 μm; **(B)** standard scale was 5 μm. **(C)** Abscisic acid content. Data indicate means ± standard deviation with three replications. Different letters for the same variety indicate significant differences between treatments as tested by LSD at *p*< 0.05. LSD, least significant difference.

In response to water scarcity, plants activate their drought response mechanisms, including the modification of stomatal morphology. As the degree of drought stress increased, notable changes were observed in the length, width, and density of the stomata (**Table 1** and **Figures 3A, B**). Under LD, MD, and SD conditions, the stomatal length and width of HN44 decreased significantly compared to the control treatment, while only SW decreased significantly for SN14 under MD and SD conditions.

**Table 1:** Effect of gradual water deficit on the leaf stomatal parameters at the abaxial surface.

Variety	Treatments	SL (μm)	SW (μm)	SD_L_ (no./mm^2^)
HN44	NW	11.97 ± 0.37^a^	4.01 ± 0.09^a^	261.6 ± 10.59^a^
	LD	10.97 ± 0.57^a^	1.67 ± 0.12^b^	180 ± 10.17^b^
	MD	8.35 ± 0.24^b^	0.93 ± 0.06^c^	166.4 ± 5.24^b^
	SD	7.90 ± 0.29^b^	0.84 ± 0.04^c^	120 ± 5.27^c^
SN14	NW	11.56 ± 0.40^b^	2.59 ± 0.20^a^	205.6 ± 9.56^a^
	LD	13.45 ± 0.54^a^	2.4 ± 0.20^a^	140 ± 7.03^b^
	MD	10.04 ± 0.38^c^	1.04 ± 0.05^b^	115.2 ± 6.1^c^
	SD	8.3 ± 0.37^d^	0.93 ± 0.06^b^	96 ± 4.30^c^

Data indicate means ± error deviation with 10 replications. Different letters for the same variety indicate significant differences between treatments as tested by LSD at p< 0.05.

SL, stomatal length; SW, stomatal width; SDL, stomatal density; NW, normal water; LD, light drought; MD, moderate drought; SD, severe drought; LSD, least significant difference.

ABA is closely associated with plant responses to environmental stress and stomatal movement. ABA content increased with increasing water stress for HN44 and SN14 (**Figure 3C**). According to the ABA content level under NW treatment, HN44 had characteristics of drought and stress resistance. The ABA contents of HN44 under LD and MD conditions were higher than those of SN14, which promoted stomatal closure in HN44 leaves; this rapidly regulated stomatal morphology in the initial stage of water shortage led to reduced Gs and transpiration and improved drought resistance and tolerance.

#### 3.1.3 Effect of gradual water deficit on WUE_i_ and RLWC

**Figure 4:**
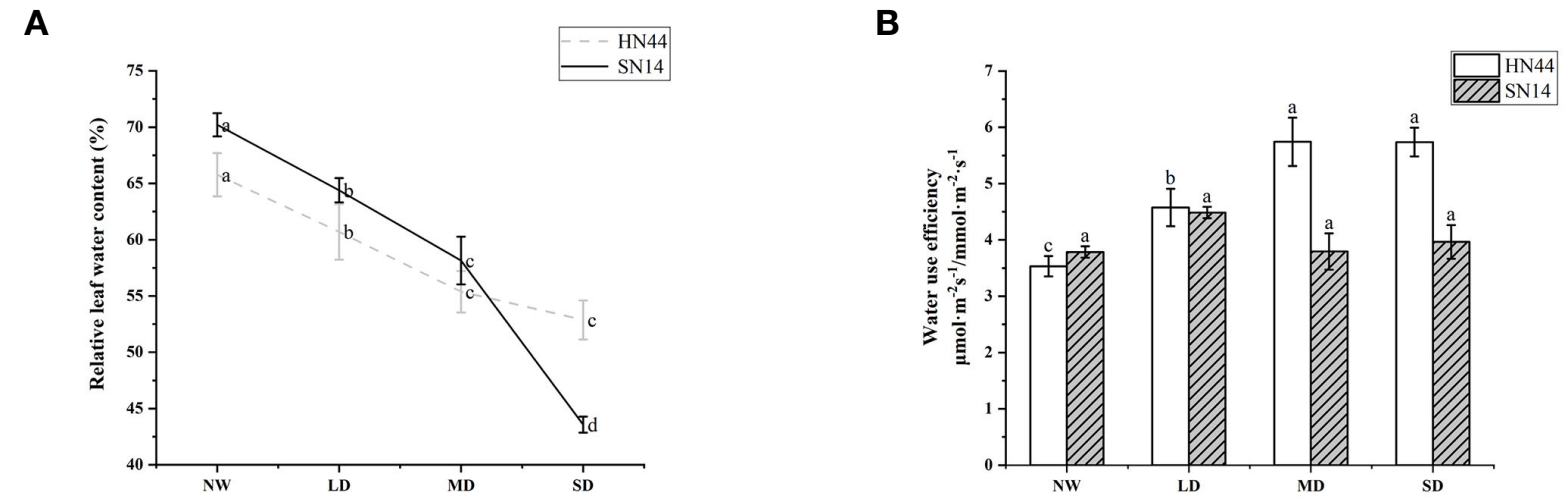
**(A)** Effects of gradual water deficit on the leaf relative water content. Data indicate means ± standard deviation with three replications. Different letters for the same variety indicate significant differences between treatments as tested by LSD at *p*< 0.05. **(B)** Effects of gradual water deficit on the instantaneous water use efficiency. Data indicate means ± standard error with five replications. Different letters for the same variety indicate significant differences between treatments as tested by LSD at *p*< 0.05. LSD, least significant difference.

Under water deficit conditions, plants actively avoid drought stress by closing the stomata, reducing transpiration, and maintaining efficient water use. With the increase of water deficit in HN44, WUE_i_ increased significantly (*p*< 0.05); under LD, MD, and SD, it increased by 28.81%, 61.30%, and 62.71% respectively, but WUE_i_ of SN14 increased insignificantly (**Figure 4B**). During the water deficit, the drought-tolerant variety HN44 showed high WUEi.

RLWC can well reflect the water condition in plant cells. With increasing severity of water deficit, RLWC of both varieties was significantly reduced (*p*< 0.05). For HN44, RLWC decreased by 7.71%, 15.80%, and 19.61% under LD, MD, and SD conditions, respectively. In comparison, SN14 showed reductions of 8.28%, 17.17%, and 37.94% under LD, MD, and SD conditions, respectively, in RLWC (refer to **Figure 4A**). In the occurrence of drought stress, the elevated ABA content of HN44 regulated the stomatal movement and morphological structure, leading to timely and effective reductions in photosynthetic indexes such as Tr, and this maintained adequate water levels and normal physiological functions within the plant.

#### 3.1.4 Effect of gradual water deficit on antioxidant enzyme activities, MDA content, and proline content

**Figure 5:**
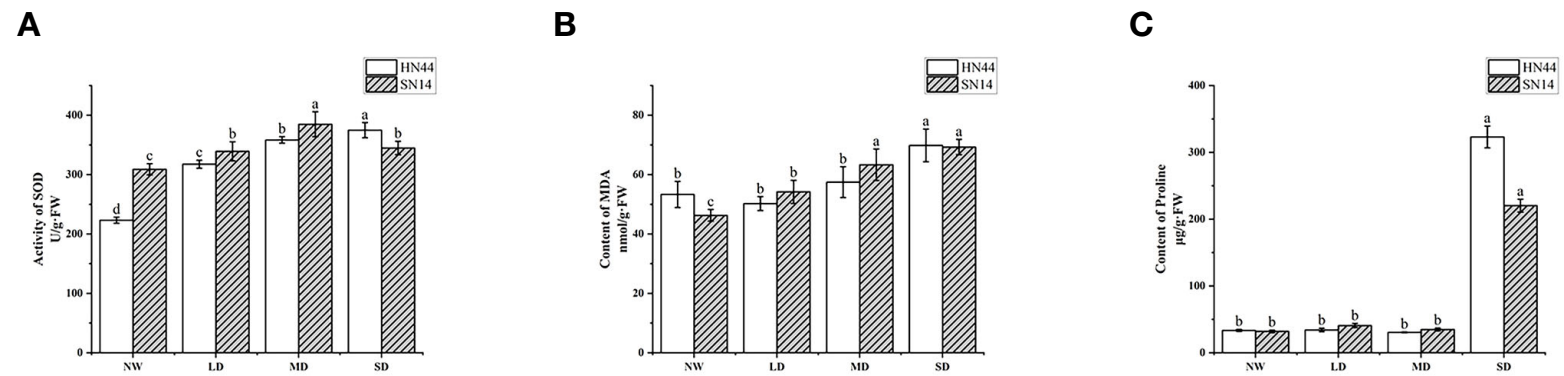
Effect of gradual water deficit on antioxidant enzyme activity and osmoregulatory substances. **(A)** The activity of SOD. **(B)** The content of MDA. **(C)** The content of proline. Data indicate means ± standard deviation with three replications. Different letters for the same variety indicate significant differences between treatments as tested by LSD at *p*< 0.05. SOD, superoxide dismutase; MDA, malondialdehyde; LSD, least significant difference.

SOD plays a pivotal role in catalyzing the dismutation of superoxide anion radicals (O_2_^−^), eliminating O_2_^−^, maintaining the balance of reactive oxygen metabolism, and protecting membrane structures. Its activity level serves as an essential indicator of plant stress tolerance. As drought stress intensified, in comparison with NW conditions, the SOD activity of HN44 increased by 42.33%, 60.65%, and 68.07% in LD, MD, and SD treatments, respectively, reaching significant differences (*p*< 0.05); the content of SN14 increased by 9.8%, 24.56%, and 11.62% in LD, MD, and SD treatments, respectively (**Figure 5A**). With the increase in the water stress degree, the increase in SOD activity of HN44 in each treatment was greater than that of SN14. The SOD content of HN44 showed a gradual upward trend, and that of SN14 showed a trend of first increasing and then decreasing. It was indicated that HN44 can maintain its balance of active oxygen metabolism and reduce the damage of free radicals to itself during drought stress. These results suggest that HN44 can maintain its reactive oxygen metabolism balance well in the process of increasing drought stress, reducing the damage of free radicals to itself. However, the activity of SOD of SN14 was inhibited severely under SD, disrupting the balance of reactive oxygen metabolism, and its ability to resist oxidative stress was weaker than that of HN44.

MDA is a significant product of plant membrane lipid peroxidation and is often used as an indicator of the degree of cell membrane lipid peroxidation, cell damage, and the intensity of plant responses to adverse conditions. The MDA content of HN44 and SN14 significantly increased under SD treatment (*p*< 0.05). Compared with NW, the MDA content of HN44 decreased by 5.81% under LD and increased by 7.74% and 30.97% under MD and SD, respectively. The MDA content of SN14 increased by 17.12%, 36.81%, and 47.45% under LD, SD, and MD treatments, respectively, compared with NW (**Figure 5B**). The increase in MDA content in HN44 under LD, MD, and SD treatments was less than that in SN14. It suggested that with the increasing drought stress, the degree of membrane damage in SN14 was greater than that in HN44.

Plants often accumulate significant amounts of proline under various adversities. Beyond its role in osmotic regulation, proline plays a crucial role in preventing excessive dehydration and cell death. In the early stage of stress, the proline content did not significantly differ from the control. However, under SD, the proline content significantly increased (*p*< 0.05). Compared with the NW, the proline content of HN44 and SN14 under SD increased by 865.85% and 587.45%, respectively (**Figure 5C**). The increase in proline content in HN44 was much higher than that in SN14, and HN44 accumulated more proline to maintain its osmotic regulation balance and tissue water retention ability, thereby enhancing its adaptability to drought stress.

### 3.2 Transcriptome analysis

#### 3.2.1 RNA-seq data revealed DEGs in soybeans under gradual water deficit conditions

**Figure 6:**
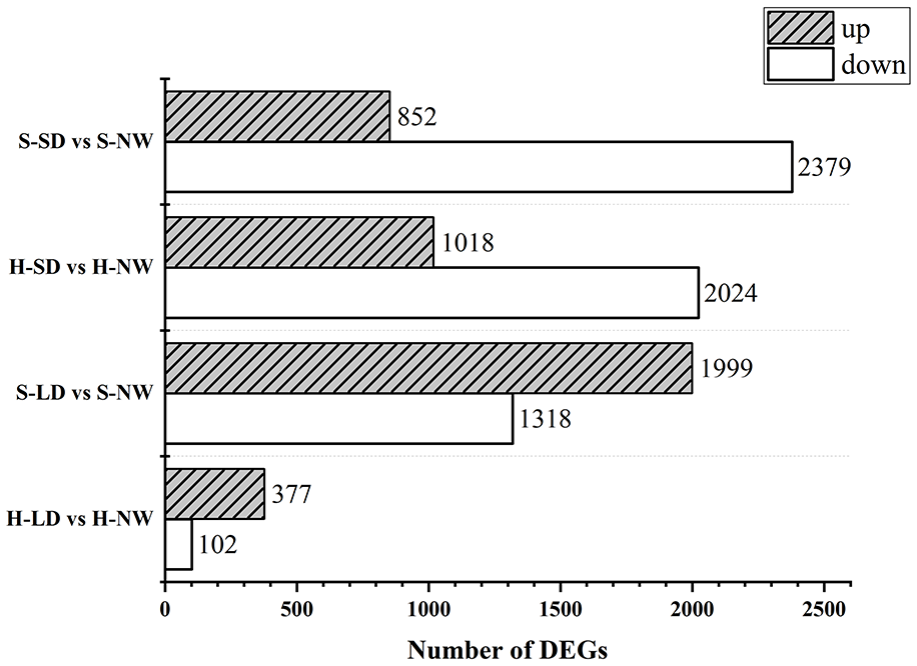
Number comparison of differentially expressed genes between both varieties under drought stress. H-NW, HN44 under normal Water; H-LD, HN44 under light drought; H-SD, HN44 under severe drought; S-NW, SN14 under normal water; S-LD, SN14 under light drought; S-SD, SN14 under severe drought. The same as below.

A total of 99.2 GB of raw data was initially obtained, and following processing with fastp (v0.23.1), 86.9 GB of clean data was obtained. In the comparison between LD and NW, 479 and 3,317 DEGs were identified in HN44 and SN14, respectively. Among these, 377 and 1,999 genes were upregulated, and 102 and 1,318 genes were downregulated in HN44 and SN14, respectively. Notably, HN44 exhibited significantly fewer DEGs under LD as compared to SN14. In the comparison between SD and NW, 3,042 and 3,231 DEGs were identified in HN44 and SN14, respectively. Among these, 1,018 and 852 genes were upregulated, and 2,024 and 2,379 genes were downregulated in HN44 and SN14, respectively (**Figure 6**).

#### 3.2.2 GO enrichment analysis of the DEGs

**Figure 7:**
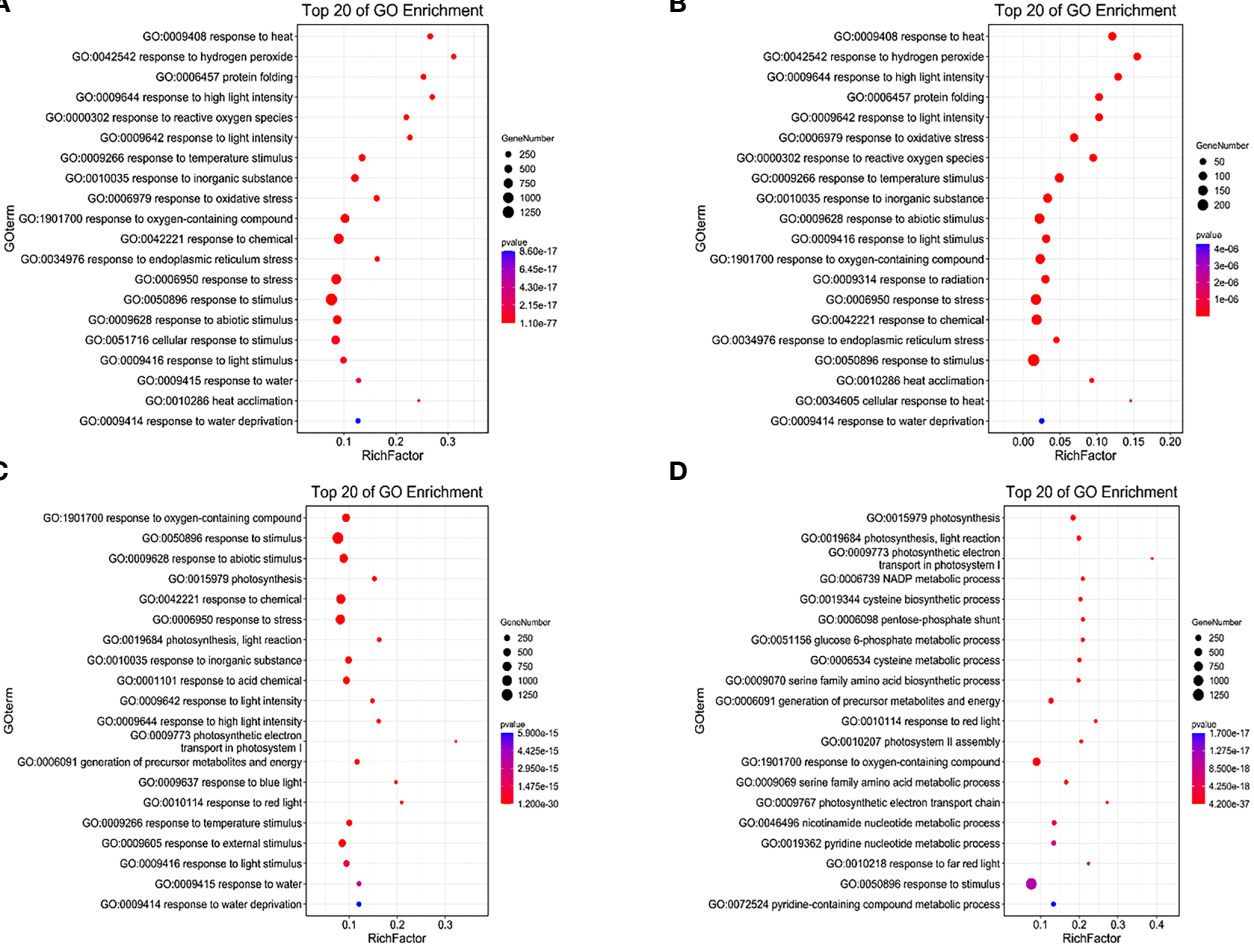
GO enrichment of differentially expressed genes between both varieties under water deficit. **(A)** H-LD *vs.* H-NW; **(B)** S-LD *vs.* S-NW; **(C)** H-SD *vs.* H-NW; **(D)** S-SD *vs.* S-NW. GO, Gene Ontology.

Under LD treatment, the DEGs of HN44 and SN14 were enriched in stress-related terms such as response to hydrogen peroxide (GO:0042542), reactive oxygen species (GO:0000302), light stimulus (GO:0009416), and signaling (GO:0007165), with most being upregulated (**Figures 7A, B**). It indicates that plants induced upregulation genes in these pathways to resist the effects of mild stress in the early stage of stress. SN14 was associated with more pathways and genes, requiring more energy consumption to resist water stress.

Under SD treatment, in addition to being enriched in the same terms as under LD, more DEGs were enriched in photosynthesis-related terms such as photosynthesis (GO:0015979), chloroplast thylakoid membrane (GO:0009535), and plastid thylakoid membrane (GO:0031976), with most being downregulated (**Figures 7C, D**). This suggests that cellular components, particularly the photosynthetic membrane system, were highly sensitive to stress conditions. As drought stress intensified, photosynthesis was inhibited, and the membrane system suffered severe damage.

#### 3.2.3 KEGG enrichment analysis of the DEGs

**Figure 8:**
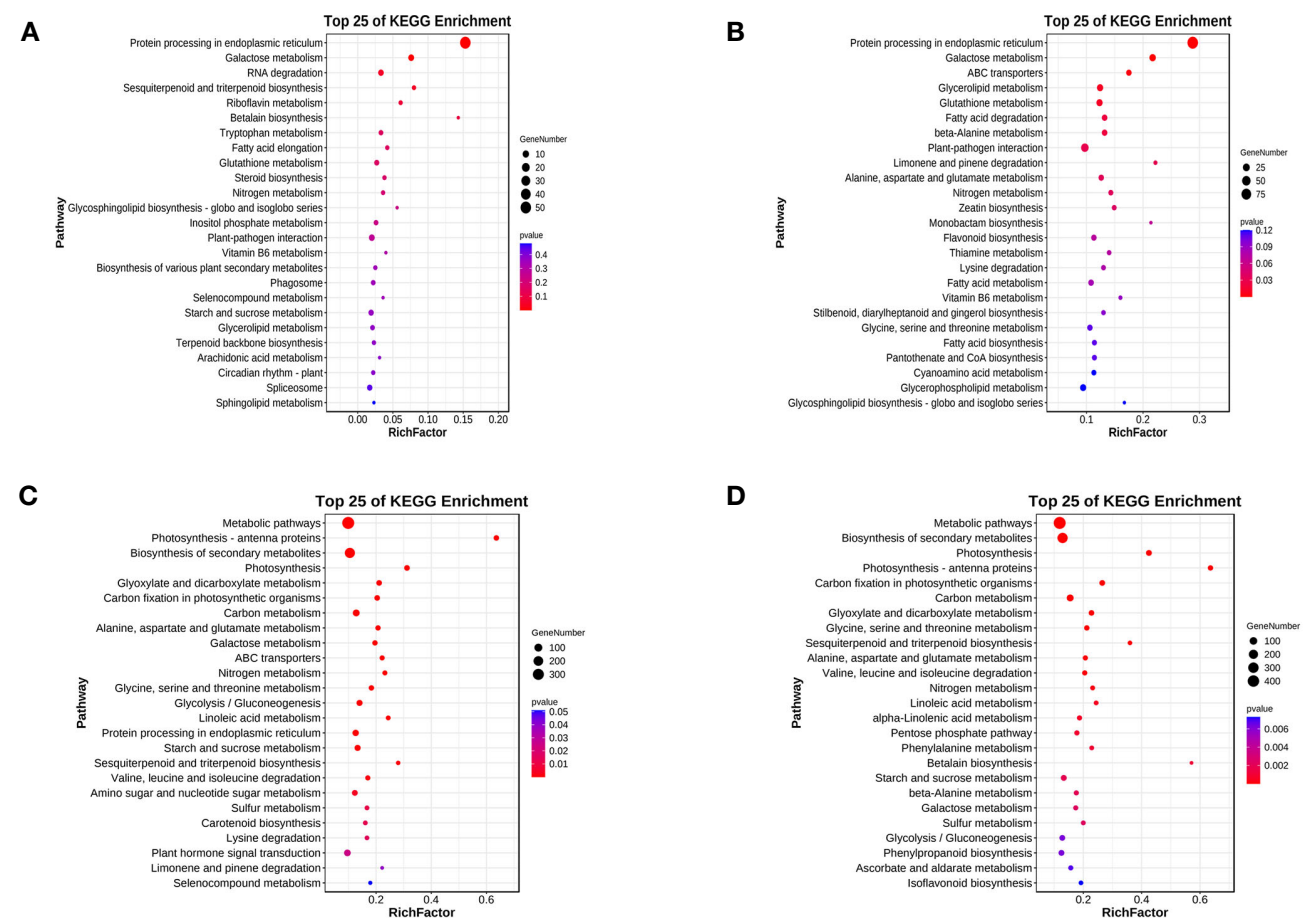
Top 25 KEGG pathway enrichment of differentially expressed genes between both varieties under water deficit. **(A)** H-LD *vs.* H-NW; **(B)** S-LD *vs.* S-NW; **(C)** H-SD *vs.* H-NW; **(D)** S-SD *vs.* S-NW. KEGG, Kyoto Encyclopedia of Genes and Genomes.

For LD treatment, HN44 and SN14 had DEGs enriched in 56 and 115 KEGG pathways, respectively, with 2 and 11 pathways showing significant enrichment (*p*< 0.05). Among these pathways, two were co-enriched in both varieties, namely, “protein processing in the endoplasmic reticulum” (ko04141) and “galactose metabolism” (ko00052) (as illustrated in **Figures 8A, B**). Under LD conditions, most DEGs enriched in “protein processing in the endoplasmic reticulum” (ko04141) were upregulated, potentially playing a crucial role in ensuring proper protein folding or positively regulating drought tolerance. Under SD treatment, both varieties exhibited co-enriched in multiple pathways, including photosynthesis-antenna proteins (ko00196), photosynthesis (ko00195), glyoxylate and dicarboxylic acid metabolism (ko00630), carbon fixation in photosynthetic organisms (ko00710), galactose metabolism (ko00052), glycolysis/gluconeogenesis (ko00010), ABC transporter proteins (ko02010), carotenoid biosynthesis (ko00906), glycine, serine, and threonine metabolism (ko00260), and other pathways related to amino acid metabolism (**Figures 8C, D**), and most of the genes showed downregulated expression, indicating that both photosynthesis and energy metabolism were differently affected under SD.

##### 3.2.3.1 Expression of differential photosynthetic pathways (ko00195)

**Figure 9:**
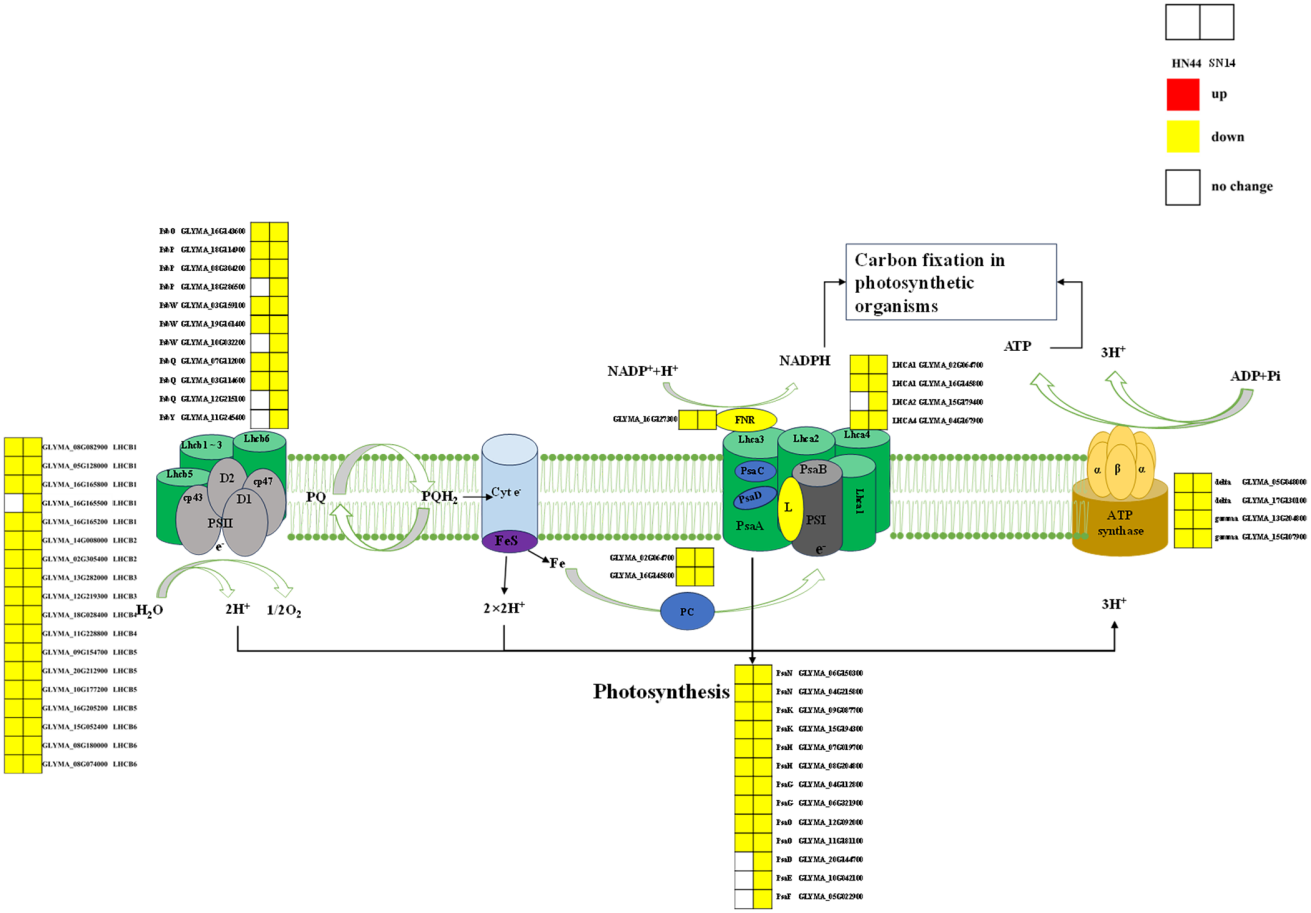
Photosynthetic pathway diagram. PSII, optical system II. PSII proteins included PsbO, PsbP, PsbQ, PsbY, and PsbW. PSI proteins included PsaD, PsaE, PsaF, PsaN, PsaK, PsaH, PsaG, and PsaO. Antenna proteins included Lhca1, Lhca2, Lhca4, Lhcb1, Lhcb2, Lhcb3, Lhcb4, Lhcb5, and Lhcb6. Type F atp enzyme protein included atp enzyme gamma, delta. Red represents the upregulated gene, yellow represents the downregulated genes, and white represents no significant change in the gene. The same as below.

Photosynthesis is a vital process responsible for the conversion of materials and energy, serving as the foundation for the survival and development of nearly all life forms on Earth. During periods of severe drought stress, both HN44 and SN14 exhibited downregulation of genes associated with the regulation of the photosynthetic pathway, as illustrated in **Figure 9**. Notably, HN44 had fewer DEGs enriched in photosystem I and photosystem II as compared to SN14, and the extent of downregulation in HN44 was also lower than that observed in SN14. These findings suggest that under drought stress, HN44 experienced less inhibition of photosynthesis in comparison to SN14, ultimately resulting in a stronger carbon assimilation capacity in HN44 when compared to SN14.

##### 3.2.3.2 Analysis of phytohormone signal transduction pathways (ko04075)

**Figure 10:**
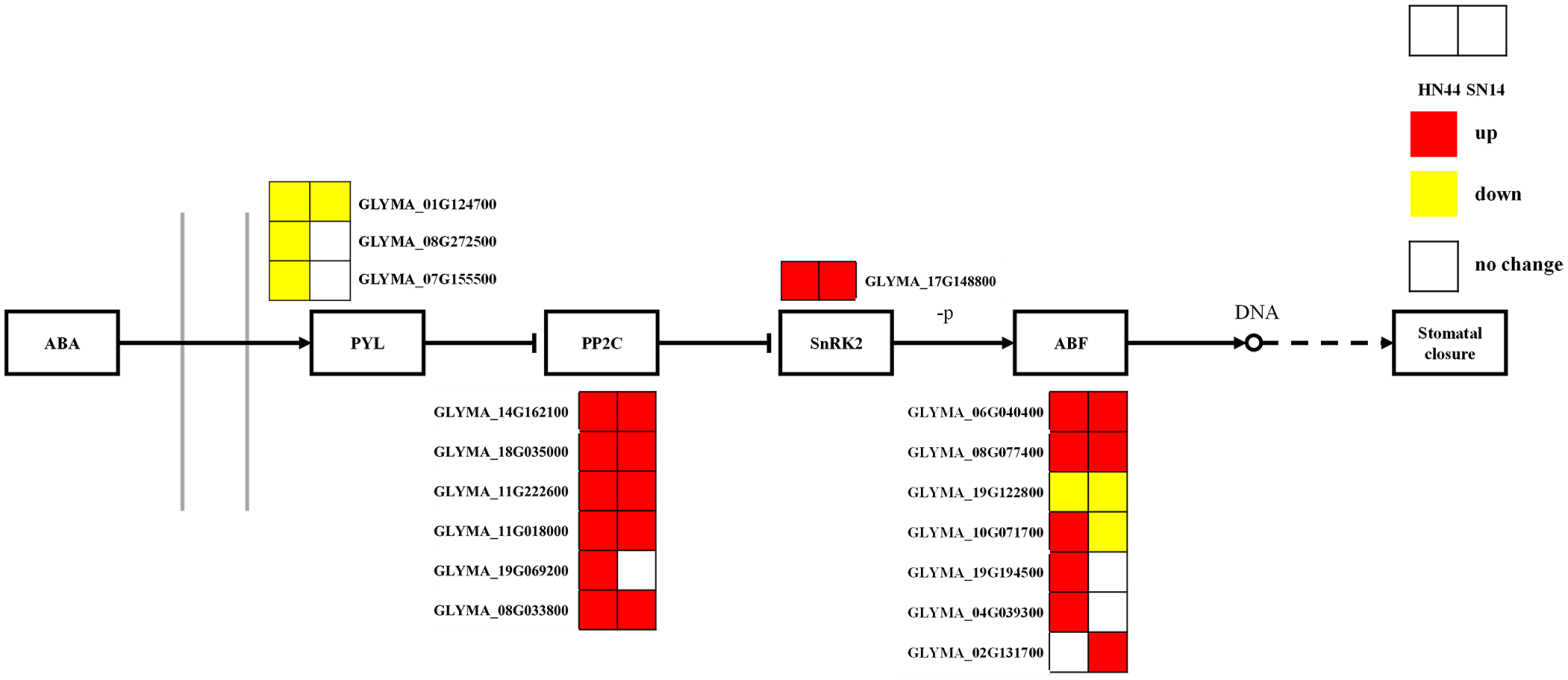
ABA-mediated signaling pathways. Red represents the upregulated gene, yellow represents the downregulated genes, and white represents no significant change in the gene. ABA, abscisic acid.

Phytohormones play a crucial role in growth, development, and adaptation to biotic and abiotic stresses. ABA plays an important role in phytohormone osmotic stress regulation, rapidly accumulating and regulating survival when plants are subjected to a variety of stresses as a stress hormone. Under severe drought stress, in both HN44 and SN14, genes regulating the ABA receptor proteins (PYR/PYL/RCAR) exhibited upregulated expression, while genes governing PP2C and SnRK2 showed downregulated expression (**Figure 10**), which allowed the regulation of the ABA response binding element ABF to promote stomatal closure, reduce water evaporation, and improve the stress tolerance of soybean.

##### 3.2.3.3 Analysis of glutathione metabolic pathways (ko00480)

**Figure 11:**
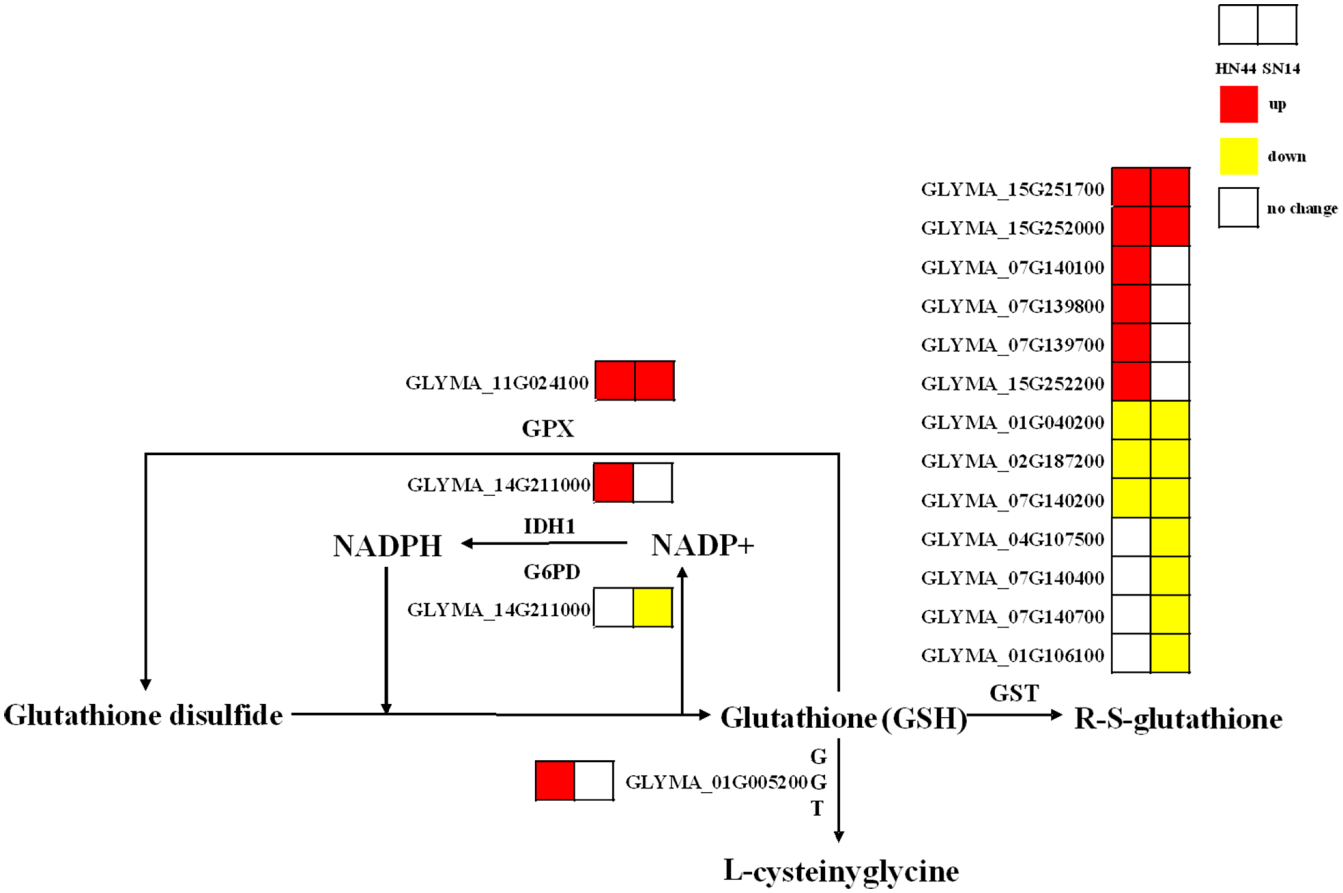
Glutathione metabolic pathway. Red represents the upregulated gene, yellow represents the downregulated genes, and white represents no significant change in the gene.

Glutathione (GSH) is a pivotal antioxidant in plants and plays an important role in various abiotic stresses. Under SD, the genes regulating glutathione peroxidase (GPX) in HN44 and SN14 were upregulated, while the genes regulating glutathione *S*-transferase (GST) were both partially upregulated and partially downregulated. In HN44, genes regulating γ-glutamyl transpeptidase (GGT) were upregulated, and the number of upregulated genes regulating GST was greater than the number of downregulated genes. SN14 had more genes downregulating GST than upregulating GST (**Figure 11**). These findings indicate that genes related to glutathione metabolism were activated, and the glutathione metabolic pathway in HN44 exhibited superior performance compared to SN14, reflecting a heightened ability to effectively mitigate ROS.

#### 3.2.4 qRT-PCR

**Figure 12:**
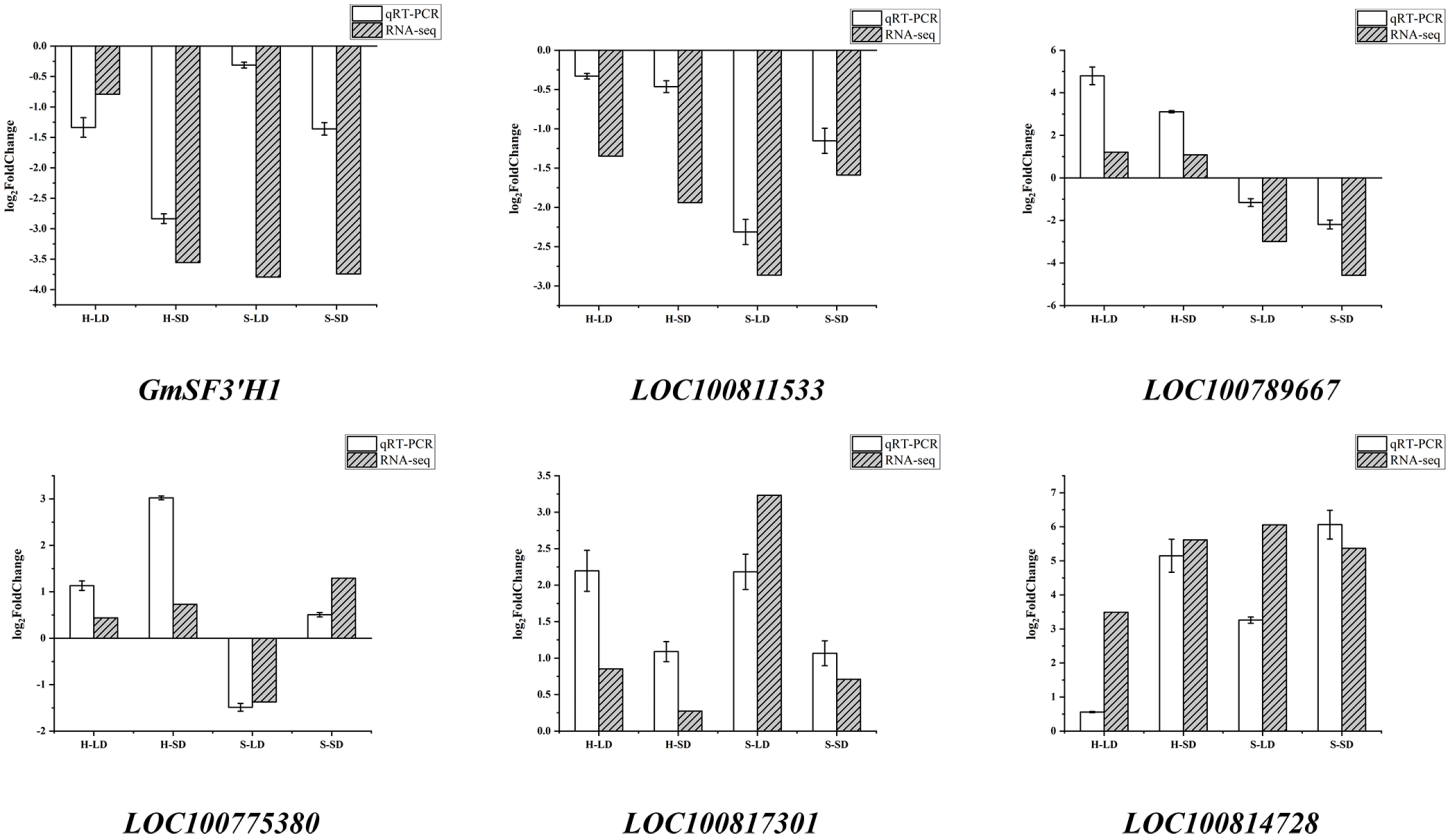
qRT-PCR verification.

Based on expression levels and related pathways, six key genes with significant differences in major drought stress response pathways were selected: the flavonoid 3′-hydroxylase gene (*GmSF3′H1*), the transcription activation factor PTI5 (*LOC100811533*), a stomatal movement-related gene (*LOC100789667*), a gene encoding SCR protein (*LOC100775380*), the phosphatidylinositol 4-kinase gamma 4 gene (*LOC100817301*), and an abscisic acid-mediated signaling pathway-related gene (*LOC100814728*). These genes were validated using qRT-PCR. As shown in **Figure 12**, the trends in gene changes were consistent with the RNA-seq results, indicating the reliability of the RNA-seq data.

#### 3.2.5 Screening of key transcription factors

Transcription factors (TFs) play important roles in regulating plant adaptation to abiotic stresses, including drought. Under LD treatment, 32 and 209 transcription factors were identified for HN44 and SN14, respectively, which indicated that SN14 activated more transcription factors to regulate pathways to resist drought stress damage. Under SD, 233 and 208 transcription factors were identified for HN44 and SN14, respectively (**Supplementary Figure 1**), with little difference in the number and type of transcription factors between the two varieties. The major transcription factor families were bHLH, ERF, WRKY, NAC, MYB, HD-ZIP, MYB_related, and bZIP, with both varieties significantly enriched in phytohormone signaling (ko04075), circadian rhythm (ko04712), and MAPK signaling pathway (ko04016).

While the number and type of transcription factors were similar between HN44 and SN14 under SD treatment, there were differences in their expression patterns. For example, the genes of the WRKY transcription factor family were partially upregulated and partially downregulated in HN44, whereas only downregulated expression was observed in SN14, and the genes of the ERF transcription factor family were expressed as mostly upregulated in HN44, and vice versa in SN14. A number of genes were screened based on the different expression patterns of the two transcription factors (**Table 2**). *LOC100808190*, *GmWRKY49*, *LOC100798375*, *LOC100804384*, *LOC100817546*, *GmbZIP4*, and *LOC100819313* genes were upregulated for expression in HN44 with no significant changes in SN14, and *GmbZIP1* was downregulated for expression in SN14 with no significant changes in HN44.

**Table 2:** Key candidate genes identified through differential expression analysis between the two soybean varieties.

Gene name	Gene description	KEGG pathway	Log_2_FoldChange		*p*-Adjust
			HN44	SN14	
*LOC100808190*	WRKY transcription factor 23	/	2.14	—	0.001201
*GmWRKY49*	WRKY transcription factor 49	MAPK signaling pathway – plant	2.25	—	1.03E-08
*LOC100798375*	WRKY transcription factor 40	/	2.46	—	0.003446
*LOC100804384*	Ethylene-responsive transcription factor ERF017	/	3.78	—	0.004281
*LOC100817546*	Ethylene-responsive transcription factor ABR1	/	2.18	—	0.001414
*GmbZIP4*	bZIP transcription factor 4	Plant hormone signal transduction	2.72	—	0.004327
*LOC100819313*	bZIP transcription factor TRAB1	Plant hormone signal transduction	2.02	—	5.77E−11
*GmbZIP1*	bZIP transcription factor 1	Plant hormone signal transduction	—	−2.26	0.000467

“/”, the gene was not enriched in KEGG pathway; “—”, the expression of this gene did not change significantly under SD treatment compared to NW treatment.

KEGG, Kyoto Encyclopedia of Genes and Genomes; SD, severe drought; NW, normal water.

## 4 Discussion

With global climate change and the increasing scarcity of water resources, drought has become one of the major abiotic stresses affecting soybean yield and quality (Chen et al., 2023). Therefore, studying the drought resistance of soybeans to improve their ability to adapt to drought is of great importance. RNA-seq is an indispensable tool for whole transcriptome differential gene expression analysis (Stark et al., 2019). This study compared the transcriptional responses of different soybean varieties under gradual water deficit conditions through RNA-seq transcriptome analysis to better understand the gene regulation patterns of soybeans under gradual water deficit conditions. Additionally, we analysed the physiological responses, in order to corroborate the factors causing differences in drought tolerance between the contrasting soybean varieties.

### 4.1 Change of physiology in soybean under gradual water deficit

As the degree of drought stress increased, both HN44 and SN14 experienced a reduction in their photosynthetic capacity, and all photosynthetic parameters were gradually reduced. However, HN44 had a greater reduction in Gs to promote stomatal closure and rapidly reduce transpiration, a greater reduction in Ci, and more CO_2_ participation in the Calvin cycle to increase HN44’s carbon assimilation to improve WUE compared with SN14. Research by McAusland et al. (2016) highlighted the importance of dynamic WUE_i_ as well as the underlying Pn and Gs as light intensity changes. Gs responded more slowly than Pn to decreasing light intensity, leading to the desynchronization of a dynamic Pn and Gs, such as when switching from high to low light, resulting in excess transpiration and reduced WUE_i_. It indicated that WUE_i_ could be improved by accelerating the rate of change in Gs (Way and Pearcy, 2012; McAusland et al., 2016). Leaves with more rapid changes in Gs often had many smaller stomata, likely due to larger surface-to-volume ratios, allowing faster solute transport to manipulate guard cells (Drake et al., 2013). With increasing stress, the stomatal length and width of HN44 were smaller than those of SN14, while the decrease in stomatal density was lower than that of SN14. HN44 rapidly changed in Gs to maintain a dynamic balance between Pn and Gs and improve instantaneous WUE_i_. As drought stress intensified, the WUE_i_ of the drought-tolerant variety HN44 gradually increased, while that of SN14 showed a wavy trend with an increase under LD followed by a decrease under MD and SD. The drought-tolerant variety was able to maintain high WUE_i_ despite being under SD, while the drought-sensitive variety showed an increase in WUE_i_ under LD followed by a decreasing trend under SD. It was consistent with the results of a study by Fletcher et al. (Fletcher et al., 2007). Drought stress triggers the accumulation of excess ROS in plants, necessitating the activation of antioxidant systems for their removal. If these excess ROS are not promptly cleared, they can damage plant tissues and accelerate plant aging, a notable symptom of which is the accumulation of MDA (Baldoni et al., 2016). In addition to activating antioxidant systems, plants also employ osmoregulatory strategies to mitigate the damage caused by drought stress (Ashraf and Foolad, 2007). In this study, as the degree of stress increased, the activity of SOD and the content of MDA in HN44 gradually increased, while in SN14, the content of MDA gradually increased and SOD activity peaked under MD conditions before subsequently declining. The proline content in both did not change significantly under LD or MD conditions but increased dramatically under SD conditions. Compared to SN14, HN44 had a lower increase in MDA accumulation and a higher increase in SOD activity and proline content. It showed that under drought stress, HN44 had superior antioxidant systems and osmoregulatory strategies to reduce the damage caused by drought stress.

### 4.2 Photosynthetic pathways under gradual water deficit

As drought stress intensified, the expression of many genes encoding photosystem I (PsaN, PsaK, PsaH, PsaG, and PsaO) and photosystem II (PsbO, PsbP, PsbQ, and PsbW) reaction center subunit proteins were downregulated in HN44 and SN14. These specific proteins had multiple functions in photosynthesis, and their downregulation under drought stress inhibited photosynthesis and reduced carbon assimilation (Calderone et al., 2003; García-Cerdán et al., 2011; Wang et al., 2020). The downregulation of genes enriched in the Pet family with ATP synthase led to a decrease in electron transport rates in PSII and PSI and a decrease in ATP synthesis, thus inhibiting plant photosynthesis (Caruso et al., 2009; Awasthi et al., 2014). The experiment identified downregulated expression of genes regulating ATP synthase activity in soybeans under drought stress (Das et al., 2016). In addition to the above co-regulated genes, SN14 also had downregulated expression of genes encoding PsaD, PsaE, PsaF, and PsbY proteins. PsaD and PsaE were important components of the PSI reduction site involved in electron transfer and binding of ferredoxin and NADP+ oxidoreductase (Scheller et al., 2001). PsaF was a peripheral subunit of PSI involved in the assembly and interaction of light-harvesting pigment proteins (Jensen et al., 2000). PsbY was important for the redox characterization of Cytb559 and might interact directly or indirectly with its heme motif. found that electron transfer on the acceptor side involving QA and QB was significantly slowed in the absence of PsbY, making *Arabidopsis* more susceptible to photoinhibition von Sydow et al. (2016). Compared to those in HN44, electron transport and photosynthesis were more strongly inhibited in SN14, potentially causing a variety of differences in photosynthetic and WUE_i_.

### 4.3 ABA-mediated signaling pathways under gradual water deficit

The PYL-PP2C-SnRK2 family is a core component of ABA signaling, with PYLs as ABA receptors and PP2C and SnPKs as important negative and positive regulators of ABA signaling, respectively. As ABA receptors, PYR/PYL/RCAR proteins can hinder the activity of PP2C. In the presence of ABA, the negative regulation mediated by PP2C is released. Subclass III SnRK2 (SRK2D/SnRK2.2, SRK2E/SnRK2.6/OST1, and SRK2I/SnRK2.3) phosphorylates ABA response elements (ABRE binding protein/ABRE binding factors (AREB/ABFs)), thereby activating the expression of many ABA response genes in an ABRE-dependent manner (Ma et al., 2009; Park et al., 2009; Huang et al., 2019). The AREB/ABF-SnRK2 pathway, as a major positive regulator of ABA/stress signaling, can directly regulate the expression of ABRE-mediated genes to cope with osmotic stress. Subclass III SnRK2 is a protein kinase that acts as an upstream control factor for AREB/ABFs through phosphorylation activity and plays a global regulatory role in the ABA/stress signaling pathway (Geiger et al., 2010). In this study, the downregulation of genes regulating ABA receptor (PYR/PYL/RCAR) proteins and the upregulation of PP2C and SnRK2 genes in HN44 and SN14 are consistent with the above results. It promotes ABF regulation of soybean stomatal closure, reduces water evaporation, and enhances soybean resistance through the ABA response binding element. Overall, HN44 was superior to SN14 in the ABA-mediated signaling pathway, with more enriched genes regulating the ABA pathway and a greater degree of upregulation. It allowed HN44 to better reduce its water loss, improve water use efficiency, and enhance its adaptability to drought.

### 4.4 Glutathione metabolic pathways under gradual water deficit

GSH is a primary antioxidant that can bind with electrophilic compounds and promote the reduction of peroxides (Li et al., 2022). In this study, under SD, upregulated genes in HN44 and SN14 regulated GPX, but the genes regulating GST were partially upregulated and partially downregulated. These enzymes collectively participated in the function of plants to clear excessive ROS in the body (Anderson and Davis, 2004; Kumar et al., 2013). Additionally, upregulated genes in HN44 regulated GGT, which can decompose extracellular GSH and provide cysteine (rate-limiting substrate) for *de novo* synthesis of GSH in cells. It was a key enzyme in glutathione metabolism and played a key role in GSH homeostasis (Zhang et al., 2005). The number of upregulated genes regulating GGT in HN44 was more than the number of downregulated genes. The number of downregulated genes regulating GST in SN14 was more than the number of upregulated genes. This suggests that the antioxidant system in SN14 was compromised under severe stress, resulting in reduced resistance to damage caused by drought stress.

### 4.5 The selection and differential analysis of key transcription factors

During long-term selection and evolution, plants have gradually developed the ability to rapidly perceive and adapt to external stress stimuli, in which the transmission of multiple adversity signals in plants plays a very important role in the process of abiotic stress in plants. The mechanisms of plant response to adversity are very complex, and the effect of improving plant resistance through the expression of single functional genes is insignificant, while transcription factors are involved in many different processes in plants, including growth, development, and stress signaling. These transcription factors have the ability to synergistically regulate multiple adversity-responsive genes or networks and can establish a complex network to regulate their metabolic responses, which is conducive to the timely response of plants to adversity stress and enhance tolerance.

In this study, we identified several members of the WRKY, ERF, and bZIP transcription factor families. These transcription factors exhibited different expression patterns, and some were involved in the ABA-mediated signaling pathway, regulating the ABA-responsive binding element ABF. Among them, WRKY and ERF differential transcription factors played more important roles in stress tolerance. The AP2/ERF family of transcription factors had multiple modes of regulation in response to drought stress, with hormonal regulation being the main mode. ABR1 was a negative regulator of ABA-regulated gene expression, and ABR1 mutants showed high sensitivity to ABA, osmotic stress, and salt stress (Pandey et al., 2005). ERF17 was able to increase iron deficiency tolerance; played a key role in photosynthesis, chlorophyll (Chl) biosynthesis, and respiratory electron transport; and was able to attenuate the effects of stress on plant photosynthesis and reduce membrane damage (Cheng et al., 2020). SlWRKY23 transcription factor in tomato was able to increase tomato tolerance to osmotic stress and salt stress by inducing the interaction of growth hormone and ethylene pathway (Singh et al., 2023).GmWRKY49 transcription factor was strongly induced by ABA, and its expression was significantly increased when exogenous ABA treatment was applied, which may act on the plant stress tolerance pathway (Zhou et al., 2008). WRKY18 and WRKY60 were able to increase plant sensitivity to ABA, osmotic stress, and salt stress, while WRKY40, WRKY18, and WRKY60 acted antagonistically to each other, and plants overexpressing WRKY40 were able to reduce the plant sensitivity to ABA and enhance the tolerance to osmotic stress and salt stress (Chen et al., 2010). These transcription factors were significantly upregulated in HN44 and downregulated in SN14, indicating that HN44 was better adapted to drought stress than SN14. In conclusion, HN44 promoted stomatal closure and reduced Gs and transpiration by regulating the upregulated expression of more ABA-responsive binding protein transcription factors to achieve efficient utilization of limited water.

## 5 Conclusion

Through a comparative analysis of the physiological characteristics of two soybean varieties under gradually increasing water deficit conditions, we observed distinct traits in the drought-tolerant variety, HN44. Notably, HN44 exhibited smaller stomata, higher stomatal density, and lower stomatal conductance and transpiration rate, as well as elevated abscisic acid content. These characteristics allowed HN44 to rapidly adapt its stomatal morphology and stomatal conductance, maintaining a dynamic equilibrium between net photosynthesis and stomatal conductance. As a result, HN44 retained higher relative leaf water content, and intrinsic water use efficiency was enhanced. Furthermore, HN44 sustained higher SOD activity and proline, reducing the accumulation of MDA and the damage caused by drought stress. Through transcriptome analysis, HN44 had fewer DEGs under LD and insensitivity to water deficit as compared to SN14. Under SD, the expression of genes encoding proteins in the photosynthesis pathway (including subunit proteins in photosystems I and II, Pet family proteins, and ATP synthase) was inhibited, resulting in the reduction of photosynthetic capacity and carbon assimilation ability and affecting growth and development. In terms of glutathione metabolism, genes regulating GPX and GGT in glutathione metabolism were upregulated, and the genes of the glutathione metabolic pathway in HN44 were activated with a stronger ability to clear ROS. The expression of genes encoding PYR/PYL/RCAR proteins in the ABA regulatory signaling pathway was downregulated, while the expression of PP2C and SnRK2 genes was upregulated. These genes activated the regulation of downstream ABFs, thereby controlling stomatal closure, reducing water loss, improving WUE, and enhancing drought resistance. The drought-tolerant variety HN44 exhibited better drought adaptability and WUE through differential expression of key genes in these major pathways. Based on the predicted differences in transcription factor expression regulation patterns between the two varieties, eight candidate drought resistance transcription factors had been identified: *LOC100808190*, *GmWRKY49*, *LOC100798375*, *LOC100804384*, *LOC100817546*, *GmbZIP4*, *LOC100819313*, and *GmbZIP1*. These genes were related to the MAPK signaling pathway and plant hormone signal transduction. The DEGs and key protectants involved in regulatory pathways identified in this study could provide important insights into the complex drought resistance mechanisms in plants. They could provide a theoretical basis for molecular breeding for high drought resistance and high water use efficiency of soybeans.

## Data availability statement

The datasets presented in this study can be found in online repositories. The names of the repository/repositories and accession number(s) can be found below: Bioproject accession number: PRJNA1011827.

## Author contributions

YX: Writing – review & editing, Data curation, Formal Analysis, Investigation, Validation, Writing – original draft. DS: Validation, Writing – review & editing. XQ: Validation, Writing – review & editing. MA: Writing – review & editing. SuW: Software, Validation, Writing – review & editing. XT: Validation, Visualization, Writing – review & editing. YJ: Conceptualization, Methodology, Project administration, Resources, Supervision, Validation, Writing – review & editing. ShW: Funding acquisition, Validation, Writing – review & editing.

## Supplementary material

Supplementary Figure 1 The number of transcription factors under water deficit.
