# Supplementary figures and images for: Physiological responses and transcriptome analysis of soybean under gradual water deficit
Source: Front Plant Sci. 2023 Oct 26;14:1269884. doi: 10.3389/fpls.2023.1269884 (PMC10639147; doi:10.3389/fpls.2023.1269884)

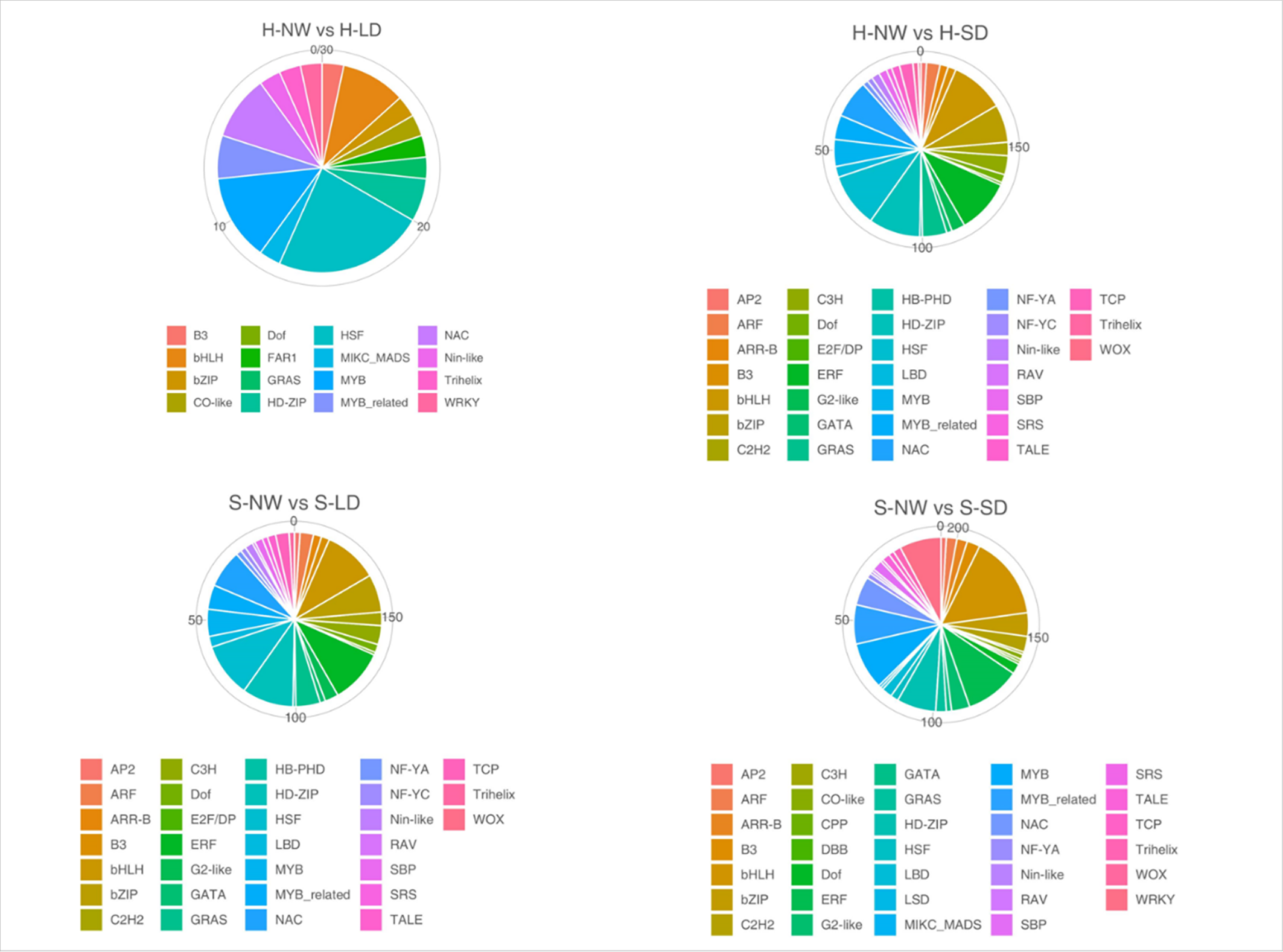

Supplement: Supplementary Figure 1 — The number of transcription factors under water deficit. [file Image_1.tif]
